# Staged caval occlusion enabling en bloc right divisional hepatectomy in two dogs with hepatocellular carcinoma

**DOI:** 10.1111/vsu.70143

**Published:** 2026-09-22

**Authors:** Kumiko Ishigaki, Kazushi Asano

**Affiliations:** ^1^ Laboratory of Veterinary Surgery, Department of Veterinary Medicine College of Bioresource Sciences, Nihon University Fujisawa Japan

## Abstract

**Objective:**

To describe staged pre‐renal caudal vena cava (CVC) occlusion before en bloc right divisional hepatectomy with segmental caval resection in two dogs with right divisional hepatocellular carcinoma (HCC) involving the CVC.

**Animals:**

Two client‐owned, 11‐year‐old neutered male dogs (Siberian Husky and Shih Tzu).

**Study design:**

Short case series.

**Methods:**

Dog 1 (Siberian Husky) had prior hemoperitoneum and biopsy‐confirmed HCC; dog 2 (Shih Tzu) had persistent liver enzyme elevations and a suspected hepatic tumor. Computed tomography (CT) showed right divisional masses (91.8 × 122.3 × 101.4 mm; 77.0 × 94.8 × 70.2 mm) with CVC compression or involvement and no pre‐existing collateral venous return. A percutaneously controlled hydraulic occluder was placed around the pre‐renal CVC. Gradual outpatient occlusion was performed over 79 and 44 days while monitoring saphenous venous pressure, renal values, and hindlimb edema. Complete CVC occlusion and collateralization were confirmed by CT before en bloc right divisional hepatectomy with segmental CVC resection.

**Results:**

Macroscopically complete resection was achieved in both dogs. Complete HCC resection was confirmed histopathologically. Morbidity was limited to transient wound exudation in dog 1 and transient ascites in dog 2. No recurrence or metastasis was identified in dog 1 during follow‐up. In dog 2, no clinical signs suggestive of recurrence or metastasis were reported. Survival after hepatectomy was 352 and 2467 days.

**Conclusion:**

Staged pre‐renal CVC occlusion may establish collateral venous return and permit planned en bloc right divisional hepatectomy with segmental CVC resection in carefully selected dogs with right divisional HCC involving the CVC.

## 1 INTRODUCTION

Hepatocellular carcinoma (HCC) is the most common primary hepatic tumor in dogs and accounts for up to 77% of primary hepatic tumors.^1^ Most canine HCCs occur as massive tumors, defined as large solitary masses arising from a single hepatic lobe.^1^ Surgical excision is considered the treatment of choice for massive HCC and is generally associated with a favorable postoperative prognosis.^1^, ^2^

Tumor location has been associated with outcome in canine HCC.^2^, ^3^ Long‐term survival following resection of left divisional tumors is typically superior to that for right divisional disease.^2^ Intraoperative complications, including severe hemorrhage, have been documented more frequently during right divisional than left divisional hepatectomy.^3^ Right divisional hepatectomy is technically challenging. Right‐sided tumors are generally more difficult to expose than left‐sided tumors. In addition, the main and accessory right hepatic veins course through the right divisional parenchyma before draining into the intrahepatic caudal vena cava (CVC).^4^ Large right divisional HCCs may obscure or incorporate these hepatic veins and the adjacent CVC, increasing the risk of vascular trauma and inadequate hemostasis during dissection. Although inflow control can reduce arterial and portal venous inflow to the liver, hemorrhage from injured hepatic veins or the intrahepatic CVC may remain substantial.

Complete resection is challenging when a large HCC arises from the right hepatic division and compresses or involves the CVC. The prognostic impact of resection completeness in canine HCC remains controversial. In some studies, margin status was not associated with long‐term prognosis.^2^, ^5^, ^6^ In others, complete resection was associated with improved survival.^7^, ^8^ Accordingly, when surgical management is pursued, complete resection should be the intended goal. For dogs with right divisional disease compressing or involving the CVC, a strategy that avoids individual right hepatic venous dissection is desirable. Such a strategy should also permit en bloc resection of the involved caval segment.

En bloc resection of HCC involving the CVC can be feasible when alternative caudal venous return is already present.^9^ In the previously described dog, a collateral vein connected the CVC to the azygos vein. This collateralization permitted CVC ligation and transection, and the hepatic mass was excised with the involved caval segment without caval reconstruction.^9^ Adequate collateral venous return may therefore allow segmental CVC resection.

Gradual pre‐renal CVC occlusion with a hydraulic occluder (HO) has also been experimentally validated in healthy dogs.^10^ In that model, the CVC was gradually occluded over 2 weeks with an occluder placed cranial to the renal veins. Collateral vessels formed, and adverse clinical signs were not detected.^10^ Based on these observations, gradual pre‐renal CVC occlusion may establish alternative venous return before definitive en bloc resection when pre‐existing collateralization is absent.

Building on previous studies,^9^, ^10^ we applied this approach in two client‐owned, 11‐year‐old castrated male dogs with right divisional HCC involving the CVC and no pre‐existing collateral venous return. The dogs were a 30.5‐kg Siberian Husky and a 9.5‐kg Shih Tzu. The objective of this short case series was to describe the history, diagnostic findings, staged pre‐renal CVC occlusion protocol, en bloc right divisional hepatectomy technique including segmental CVC resection, and outcomes in these two dogs.

## 2 MATERIALS AND METHODS

### 2.1 Case selection and preoperative planning

This short case series included two client‐owned dogs with large right divisional hepatic masses compressing or involving the CVC. Both dogs underwent staged pre‐renal CVC occlusion followed by en bloc right divisional hepatectomy with segmental CVC resection. Both dogs lacked pre‐existing collateral venous return on contrast‐enhanced computed tomography (CT), and hepatocellular carcinoma (HCC) was confirmed histopathologically. Case histories and prior medical records were reviewed. Preoperative planning included physical examination, complete blood count (CBC), serum chemistry, thoracoabdominal radiography, abdominal ultrasonography, and contrast‐enhanced CT. CT was used to characterize the hepatic mass, assess CVC involvement and collateral venous return, screen for distant metastasis, and plan staged occlusion and definitive en bloc resection.

### 2.2 Surgical technique

#### 2.2.1 First‐stage surgery

Under general anesthesia, a percutaneously controlled HO was placed around the CVC between the liver and right adrenal region and connected to a subcutaneous port (Figure 1). In dog 1, the HO was a silicone vascular occluder (lumen diameter 8 mm; cuff width 7 mm; cuff thickness 2 mm; Silicone vascular occluder, Medical Agent Co., Kyoto, Japan). In dog 2, the HO was an artificial urethral sphincter occluder (8 × 14 mm; Norfolk Vets Products Inc., Skokie, Illinois). After HO placement and before abdominal closure, intraoperative test inflation was performed by injecting sterile saline into the port and then withdrawing it. During test inflation and deflation, saphenous venous pressure was measured through an 18‐gauge over‐the‐needle catheter placed in the right saphenous vein. The catheter was connected to a fluid‐filled disposable pressure transducer system (Blood Pressure Monitoring Kit, Nihon Kohden Co., Tokyo, Japan), which was connected to a multiparameter physiologic monitor (Bedside Monitor, Nihon Kohden Co.). The transducer was zeroed to atmospheric pressure at the level of the right atrium before each measurement. Saphenous venous pressure was recorded before saline injection, after test inflation, and after saline withdrawal.

**FIGURE 1:**
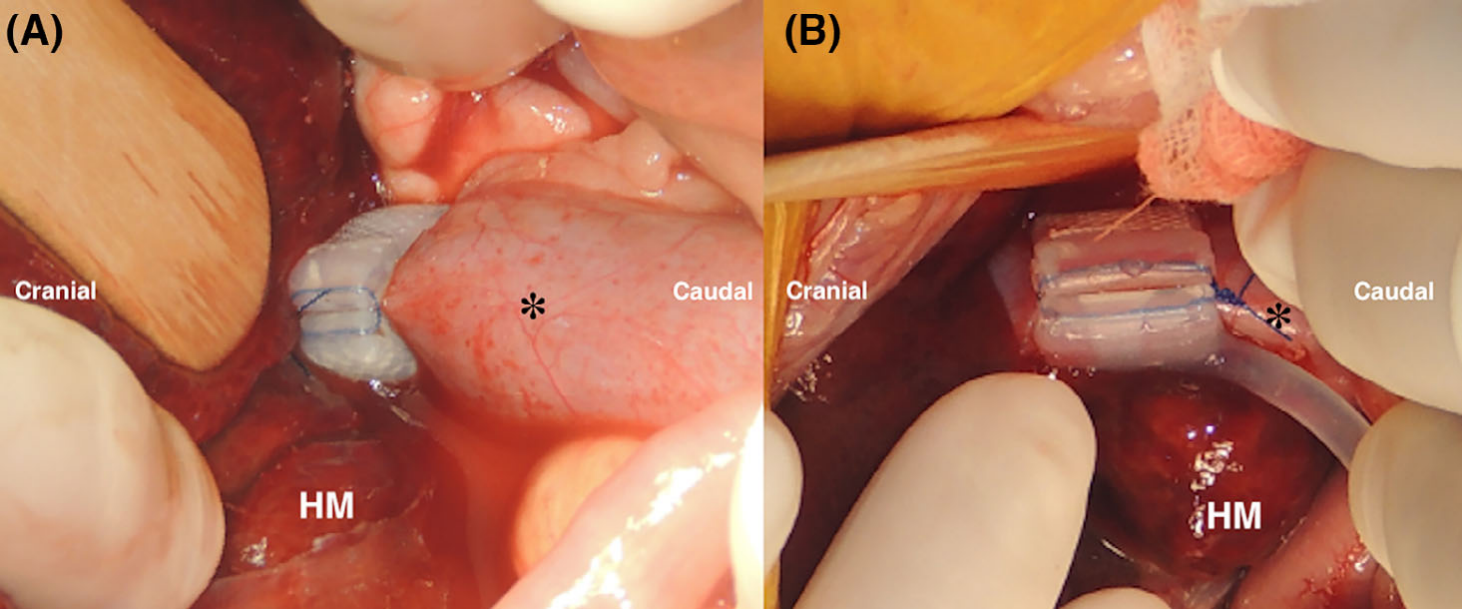
Placement of a percutaneously controlled hydraulic occluder (HO) encircling the caudal vena cava (CVC) during the first‐stage surgery. The HO was placed between the liver and right adrenal region. (A) Dog 1: Silicone vascular occluder (lumen diameter 8 mm, cuff width 7 mm, cuff thickness 2 mm). (B) Dog 2: Artificial urethral sphincter occluder (8 × 14 mm). HM, hepatic mass; Asterisk, CVC.

#### 2.2.2 Postoperative management after the first‐stage surgery

At outpatient inflation visits, small aliquots of sterile saline were injected into the subcutaneous port to gradually occlude the CVC. Measurements were performed in conscious dogs positioned in right lateral recumbency. Saphenous venous pressure was measured from the left saphenous vein using the same catheter–transducer–monitor system and zeroing procedure described for intraoperative measurements. Pressure was recorded before injection and after each incremental saline injection. Hindlimb edema and blood urea nitrogen (BUN) and creatinine (Cr) concentrations were monitored throughout the staged occlusion period. No predetermined saphenous venous pressure target or cutoff for saline withdrawal was established; additional inflation was based on the pressure response and the absence of hindlimb edema or clinically relevant renal dysfunction. Saline would have been withdrawn if an abrupt excessive increase in saphenous venous pressure or clinical or laboratory evidence of impaired caudal venous return was observed. Saphenous venous pressure was used as a patient‐specific monitoring variable and as a trigger for CT evaluation. Complete caval occlusion and collateral venous return were confirmed by CT before the second‐stage surgery. The second‐stage surgery was scheduled 1–3 months after the first‐stage surgery.

#### 2.2.3 Second‐stage surgery

Under standard general anesthesia, vascular access was established via a right jugular central venous catheter and right femoral arterial and venous catheters for continuous pressure monitoring. An abdominal Mercedes incision, consisting of a cranial midline celiotomy with bilateral paracostal extensions, was performed (Figure 2A). Adhesions around the HO were released (Figure 2B), the HO was mobilized from the CVC, and the occluded CVC was double‐ligated and transected (Figure 2C). Then, the HO system was removed. The right divisional Glissonian pedicle, including the right portal branch and right hepatic arteries and ducts, was ligated en bloc and transected (Figure 2D). Dissection of the right triangular ligaments and retroperitoneal membranes isolated the right hepatic division. The CVC cranial to the right hepatic division was double ligated and transected (Figure 2E). En bloc removal of the right hepatic division, including the mass and the involved caval segment, was then completed (Figure 2F). Central venous, femoral venous, and femoral arterial pressures were recorded at predefined time points, including before and after HO removal, after CVC ligation and transection, and after en bloc resection. Abdominal incision was closed routinely.

**FIGURE 2:**
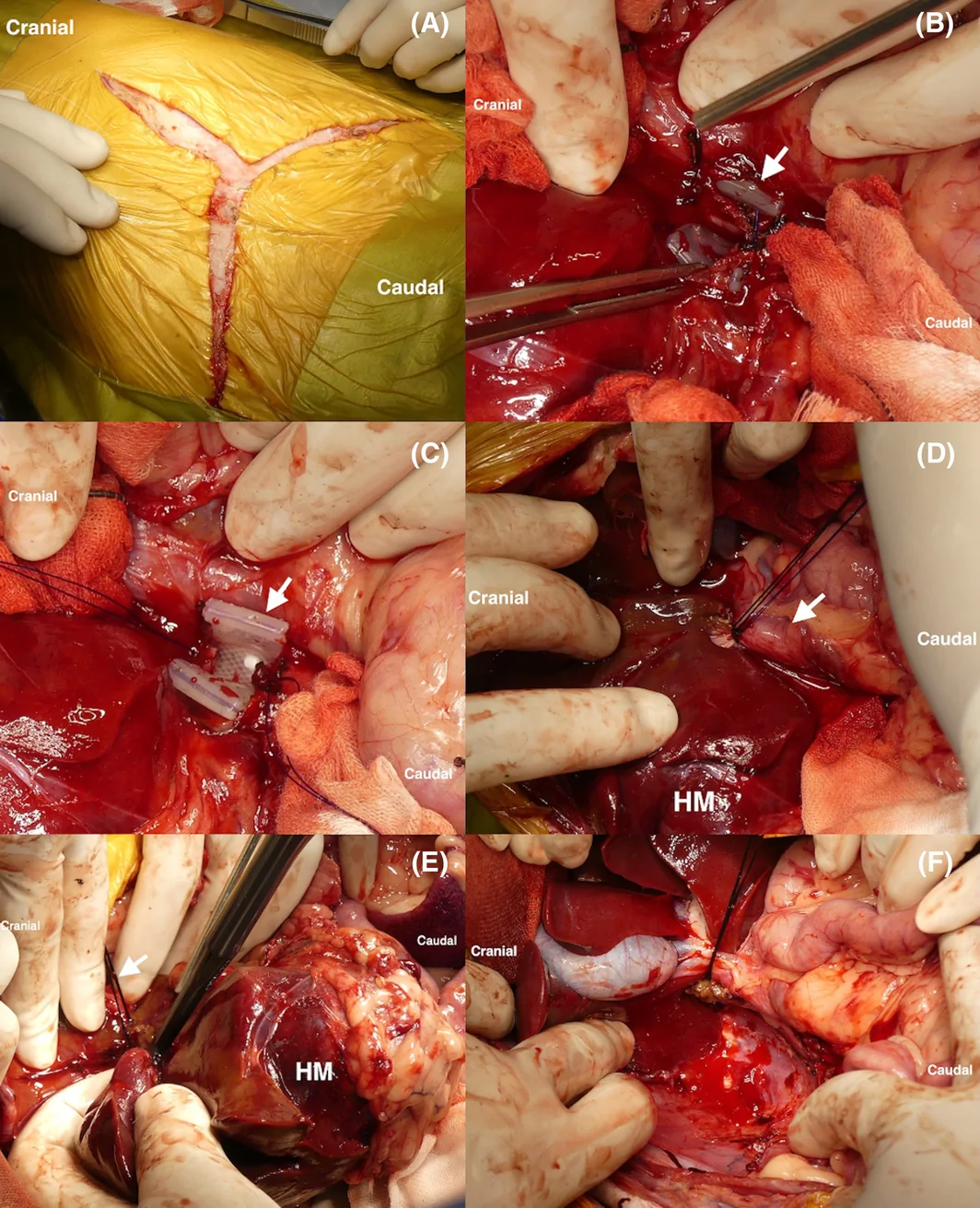
Second‐stage surgery in dog 2. (A) Abdominal Mercedes incision (cranial midline celiotomy with bilateral paracostal incisions). (B) Release of the hydraulic occluder (HO) that was implanted during the first‐stage surgery. White arrow indicates the HO. (C) Transection of the occluded caudal vena cava (CVC). White arrow indicates the HO. (D) Ligation of the right divisional Glissonian pedicle including the right hepatic arteries and ducts and right portal branch. White arrow indicates portal vein; HM, hepatic mass. (E) Ligation and transection of the CVC cranial to the right hepatic division. Two coated braided nylon sutures were passed around the CVC cranial to the right hepatic division using a right‐angle forceps. White arrow indicates the sutures; HM, hepatic mass. (F) The gross findings after en bloc resection of right hepatic division including the segmental CVC.

### 2.3 Immediate postoperative management

Immediately after the second‐stage surgery, dogs received routine intensive care including invasive pressure monitoring, IV fluids, and multimodal analgesia. Cross‐matched whole blood was prepared before the second‐stage surgery in both dogs. Both dogs had a lower packed cell volume (PCV) before the second‐stage surgery than before the first‐stage surgery. Substantial hemorrhage was anticipated during right divisional hepatectomy with segmental CVC resection, particularly because severe adhesions around the HO were expected. No fixed PCV threshold was used for transfusion. The decision to administer or continue perioperative transfusion was based on multiple factors. These included the preoperative PCV trend, anticipated surgical complexity and hemorrhage risk, estimated or observed intraoperative blood loss, serial CBC or PCV measurements, arterial blood pressure and other anesthetic/perfusion variables, and postoperative clinical status. Antiplatelet therapy (clopidogrel; Plavix, Sanofi K.K., Tokyo, Japan) was used in dog 2 after the first‐stage surgery. Laboratory testing and abdominal ultrasonography were performed as indicated to detect complications.

### 2.4 Follow‐up after the second‐stage surgery

During hospitalization, clinical status and indicated laboratory/imaging tests were monitored to detect complications. After discharge, follow‐up information was obtained from the referring veterinarians and owners. When performed by the referring veterinarians, follow‐up diagnostics, including thoracic and abdominal radiography and abdominal ultrasonography, were recorded. Survival time was calculated from the date of the second‐stage surgery to death.

## 3 RESULTS

### 3.1 Dog 1

Dog 1 was an 11‐year‐old castrated male Siberian Husky weighing 30.5 kg. A large right divisional hepatic mass and hemoperitoneum were identified at the referring hospital. Exploratory surgery at the referring hospital yielded incisional biopsies only, and histopathology confirmed HCC. The perioperative metrics of dog 1 are summarized in Table 1. Baseline CBC values were within reference intervals; serum chemistry revealed increased liver enzymes (Table 2). CT showed a large mass (91.8 × 122.3 × 101.4 mm) arising from the right division with marked CVC involvement and no collateral venous return (Figure 3A). Staged caval occlusion was planned to facilitate en bloc resection given CVC involvement without collateral flow.

**TABLE 1:** Key perioperative metrics in both dogs.

Metric	Dog 1	Dog 2
Bodyweight (kg)	30.5	9.5
Tumor size on CT (mm)	91.8 × 122.3 × 101.4	77.0 × 94.8 × 70.2
Interval: first‐ to second‐stage (days)	79	44
Outpatient saline injections (n)	2	3
CT collateralization before second‐stage	Azygos	Azygos + superficial caudal epigastric
Caval thrombus before second‐stage	Large pre‐renal (removed)	None (clopidogrel administered)
Operative time (min)	121	120
Complications	Transient wound exudation	Transient ascites
Completeness of resection	Complete	Complete
Survival after second‐stage (days)	352	2467

Abbreviation: CT, computed tomography.

**TABLE 2:** Selected laboratory values before each stage surgery in both dogs.

Variables	Unit	Dog 1		Dog 2		Reference interval
		Before first	Before second	Before first	Before second	
PCV	%	39	36	44	39	37–55
Plt	10^3^/μL	404	419	381	693	200–500
AST	U/L	20	18	90	49	0–50
ALT	U/L	421	206	1418	2119	10–100
ALP	U/L	2022	2329	420	641	23–212
GGT	U/L	19	25	21	10	0–8
BUN	mg/dL	16	25	18	32	7–27
Cr	mg/dL	0.6	1.2	1.1	0.8	0.5–1.8

Abbreviations: ALP, alkaline phosphatase; ALT, alanine aminotransferase; AST, aspartate aminotransferase; BUN, blood urea nitrogen; Cr, creatinine; GGT, gamma‐glutamyl transferase; PCV, packed cell volume; Plt, platelet count.

**FIGURE 3:**
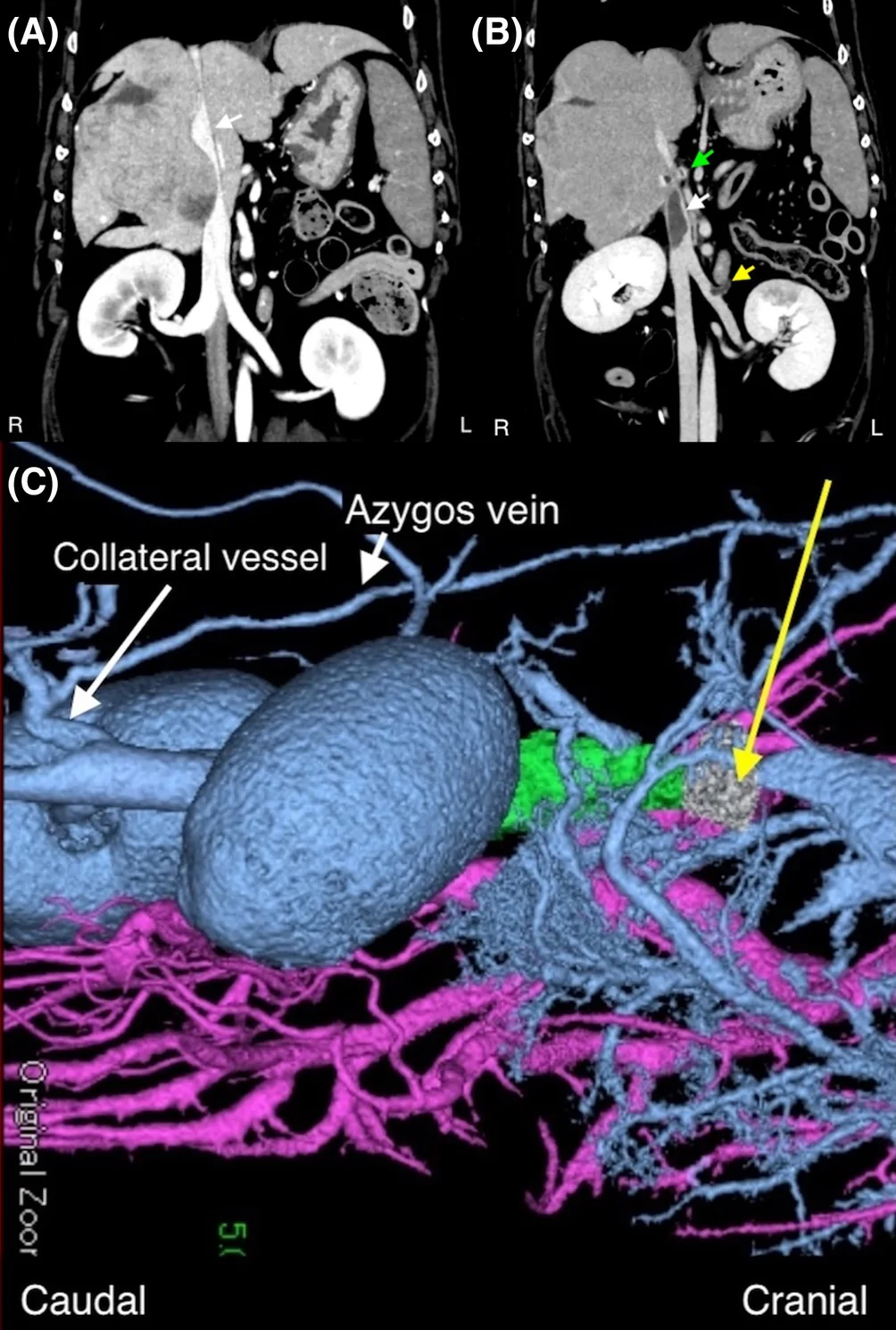
Computed tomography (CT) findings of dog 1. (A) Coronal image before the first‐stage surgery. A large right divisional hepatic mass compressed the CVC. White arrow indicates the caudal vena cava (CVC). (B) Coronal image before the second‐stage surgery. The hydraulic occluder (HO, green arrow) completely occluded the CVC, and the thrombus (white arrow) was detected in the pre‐renal CVC near the HO. In addition, another thrombus developed in the left phrenicoabdominal vein (yellow arrow). (C) Three‐dimensional (3D) reconstruction of CT angiography before the second surgery. On CT angiography, the CVC was completely occluded by the HO (yellow arrow), and the collateral vessels developed from the CVC to the azygos vein.

On day 14 after first evaluation, the placement of the HO was completed without any complications in the first‐stage surgery. The intraoperative saphenous venous pressure was 7 mmHg before HO placement. After HO placement, the venous pressure increased to 30 mmHg with injection of 0.4 mL sterile saline into the port and decreased to 8 mmHg after thorough withdrawal of the saline.

Details of the staged occlusion timeline are provided in Table 3. On day 23 after the first‐stage surgery, no hindlimb edema was observed and renal values did not indicate impaired caudal venous return. Saphenous venous pressure increased from 10 to 19 mmHg after injection of 0.2 mL saline. On day 47, pressure increased from 9 to 19 mmHg after injection of an additional 0.3 mL saline, resulting in a total retained volume of 0.5 mL. On day 79, preoperative CT prior to second‐stage surgery revealed complete occlusion of the CVC and collateral formation from the CVC to the azygos vein (Figure 3B,C). Additionally, a large thrombus was found in the CVC immediately caudal to the HO placement and a small thrombus in the left phrenicoabdominal vein.

**TABLE 3:** Timeline of staged pre‐renal CVC occlusion and saphenous venous pressure monitoring.

Dog	Time point	Day after first‐stage surgery (days)	Saline injected at visit (mL)	Total saline retained in occluder (mL)	SVP before/after injection (mmHg)	Renal values (mg/dL)		Hindlimb edema
						BUN	Cr	
#1	First surgery (intraoperative test inflation)	0	0.4	0 (after withdrawal)	7 → 30 → 8	16	0.6	NA
	Outpatient inflation 1	23	0.2	0.2	10 → 19	27	0.9	No
	Outpatient inflation 2	47	0.3	0.5	9 → 19	NA	NA	No
	Preoperative CT+ second surgery	79	NA	0.5	NA	25	1.2	No
#2	First surgery (intraoperative test inflation)	0	0.8	0 (after withdrawal)	5 → 73 → 4	18	1.1	NA
	Outpatient inflation 1	6	0.1	0.1	10 → 20	12	0.8	No
	Outpatient inflation 2	22	0.1	0.2	13 → 32	30	0.7	No
	Outpatient inflation 3	30	0.6	0.8	18 → 18	25	1.0	No
	Preoperative CT+ second surgery	44	NA	0.8	NA	32	0.8	No

*Note*: Saphenous venous pressure is reported as before injection → after injection. For intraoperative test inflation, values are reported as before injection → after test inflation → after saline withdrawal. Total saline retained in the occluder indicates the volume remaining in the occluder after completion of each visit.

Abbreviations: CVC, caudal vena cava; CT, computed tomography; Intraop, intraoperative; NA, not applicable; SVP, saphenous venous pressure.

In the second‐stage surgery, the intra‐abdominal adhesions around the HO were severe. The cranial caval and femoral venous pressures were 3 and 16 mmHg, respectively, and the femoral arterial pressure was 165/90/66 mmHg (systolic/mean/diastolic pressure) before en bloc resection of the right hepatic division. The large caval thrombus was removed immediately after removal of the HO. En bloc resection of the right hepatic division including the mass and CVC was completed, so macroscopically complete resection was achieved (Figure 4). The cranial caval and femoral venous pressures were 2 and 19 mmHg, respectively, and the femoral arterial pressure was 134/109/88 mmHg (systolic/mean/diastolic pressure) after en bloc resection. The operative time was 121 min.

**FIGURE 4:**
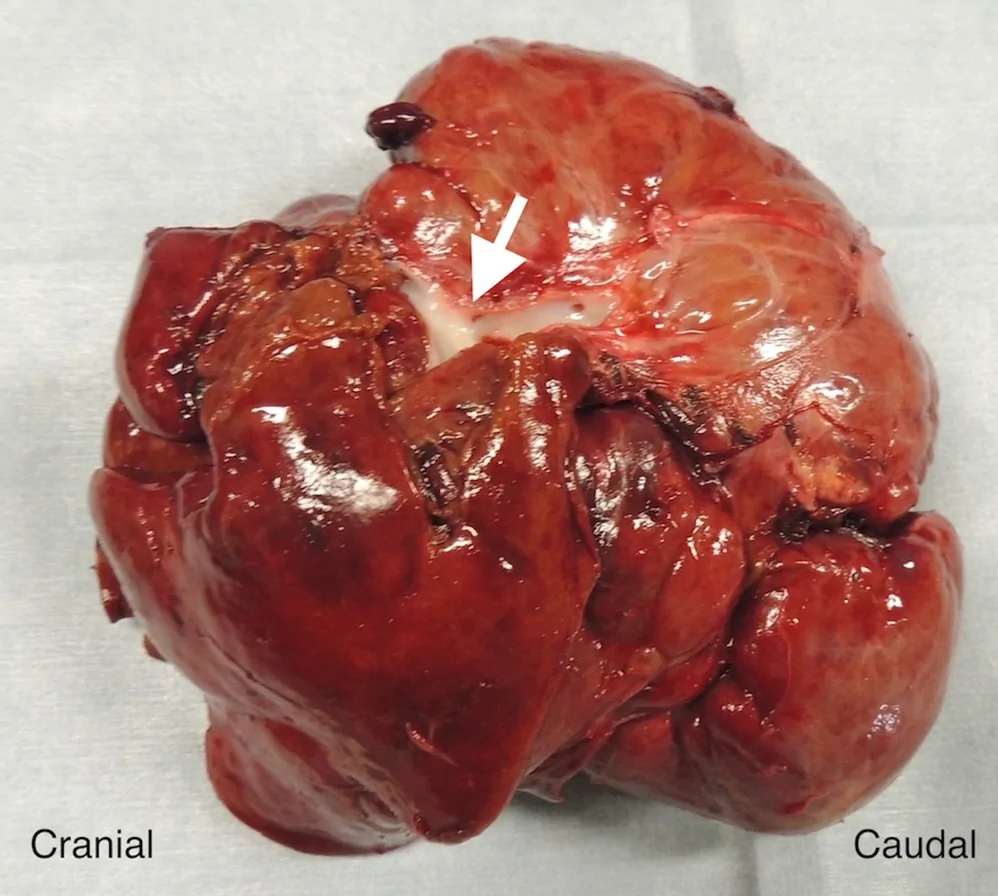
The resected mass of dog 1. White arrow indicates the sagittal section of the caudal vena cava (CVC).

Recovery from anesthesia was uneventful. A 200‐mL perioperative blood transfusion was administered because of the preoperative PCV trend and anticipated hemorrhage risk associated with right divisional hepatectomy, segmental CVC resection, and severe adhesions. The mass weighed 580 g (1.9% bodyweight). HCC with complete resection was confirmed histopathologically. The dog resumed eating on postoperative day 2; transient wound exudation resolved during hospitalization. Additionally, BUN (11 mg/dL) and Cr (0.8 mg/dL) remained within the reference intervals.

Because of the long travel distance, the dog remained hospitalized until suture removal on postoperative day 22 and was discharged thereafter. Bodyweight at discharge was 28.0 kg, and the owner reported normal appetite and activity without analgesics. At discharge on postoperative day 22, aspartate aminotransferase (AST, 29 U/L) was within the reference interval, whereas alanine aminotransferase (ALT, 265 U/L), alkaline phosphatase (ALP, 2485 U/L), and gamma‐glutamyl transferase (GGT, 11 U/L) remained increased, indicating persistent postoperative hepatobiliary enzyme elevation. Quantitative post‐discharge liver enzyme values were not available for review; therefore, laboratory follow‐up for dog 1 at our hospital was limited to postoperative day 22. Follow‐up by the referring veterinarian included periodic blood testing, thoracic and abdominal radiography, and abdominal ultrasonography. Based on the imaging examinations, the referring veterinarian reported no evidence of local recurrence or thoracic or abdominal metastasis. The dog died of unknown cause 352 days after the second‐stage surgery (445 days after the first evaluation).

### 3.2 Dog 2

Dog 2 was an 11‐year‐old castrated male Shih Tzu weighing 9.5 kg. The dog was evaluated for persistent elevation of serum liver enzymes and a large right divisional hepatic mass identified before referral. The perioperative metrics of dog 2 are summarized in Table 1. CBC values were within reference intervals; serum chemistry showed increased liver enzymes (Table 2). CT showed a mass (77.0 × 94.8 × 70.2 mm) from the right division with marked CVC involvement and no collateral return (Figure 5A). Staged caval occlusion was planned to facilitate en bloc resection given CVC involvement without collateral flow.

**FIGURE 5:**
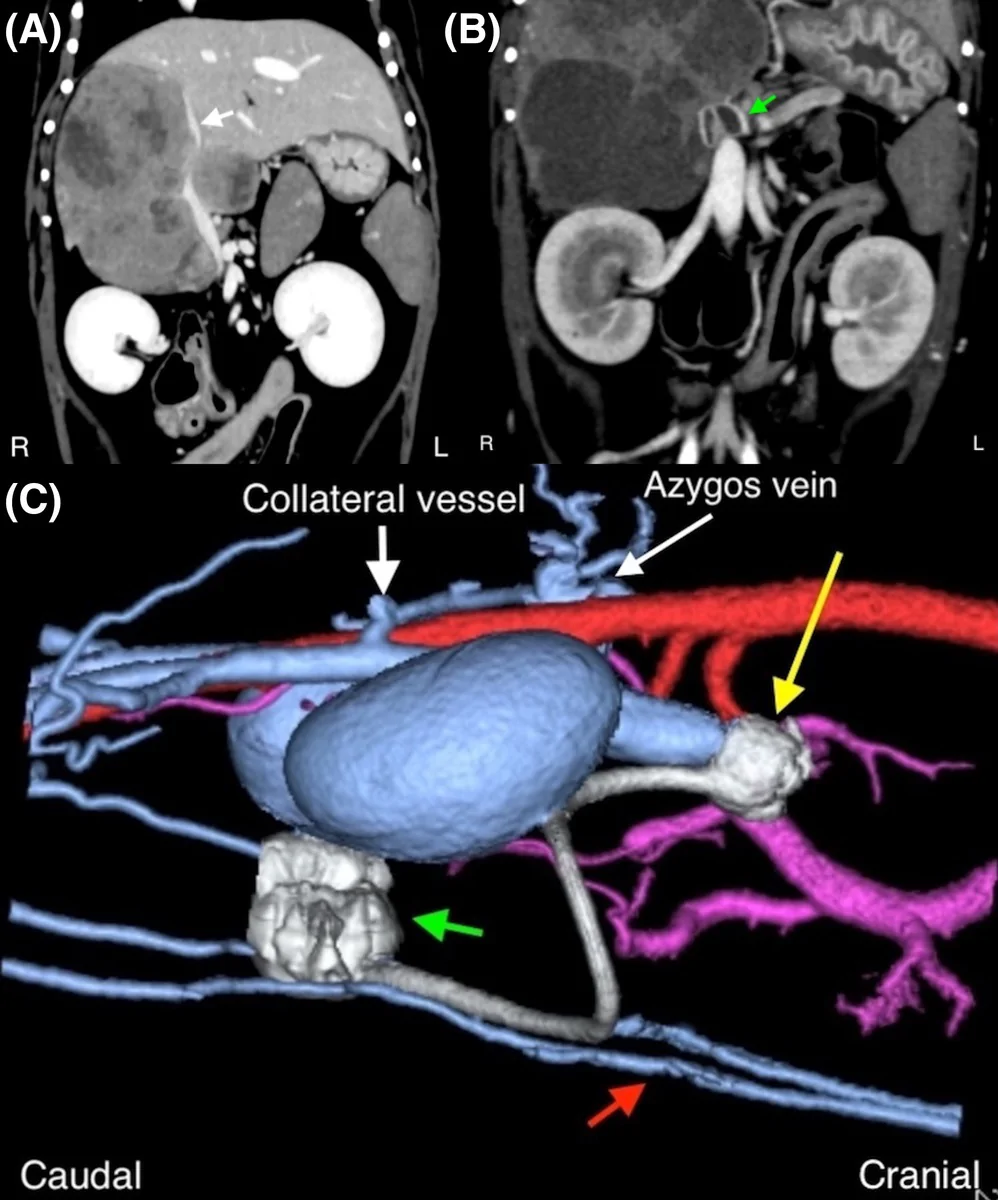
Computed tomography (CT) findings of dog 2. (A) Coronal image before the first‐stage surgery. A large right divisional hepatic mass involved the caudal vena cava (CVC). White arrow indicates the CVC. (B) Coronal image before the second‐stage surgery. The hydraulic occluder (HO, green arrow) completely occluded the CVC, and no thrombus was detected. The dog underwent clopidogrel administration after the first‐stage surgery. (C) Three‐dimensional (3D) reconstruction of CT angiography before the second surgery. On CT angiography, the CVC was completely occluded by the HO (yellow arrow), and the collateral vessels developed from the CVC to the azygos vein and superficial caudal epigastric veins (red arrow). Green arrow indicates the subcutaneous port.

The placement of the HO was completed without any complications in the first‐stage surgery. The intraoperative saphenous venous pressure was 5 mmHg before HO placement. After the HO placement, the venous pressure increased to 73 mmHg with injection of 0.8 mL sterile saline into the port and decreased to 4 mmHg after thorough withdrawal of the saline.

Beginning on postoperative day 3, clopidogrel sulfate (2.9 mg/kg, orally once daily) was administered. Details of the staged occlusion timeline are provided in Table 3. Outpatient inflations were performed on days 6, 22, and 30 after the first‐stage surgery. Saphenous venous pressure responses were 10–20 mmHg, 13–32 mmHg, and 18–18 mmHg, respectively, with a final retained volume of 0.8 mL. No hindlimb edema was observed, and Cr remained within the reference interval throughout the staged occlusion period. Clopidogrel was withheld beginning 2 days before the preoperative CT and second‐stage surgery and was not administered on the day of the procedure. On day 44, preoperative CT prior to second‐stage surgery revealed complete occlusion of the CVC, and collateral formation of venous return to the azygos and superficial caudal epigastric veins (Figure 5B,C). Additionally, no thrombosis was detected in the CVC.

In the second‐stage surgery, the intra‐abdominal adhesions around the HO were severe (Figure 6), similar to dog 1. The cranial caval and femoral venous pressures were 2 and 13 mmHg, respectively, and the femoral arterial pressure was 140/95/78 mmHg (systolic/mean/diastolic pressure). No subjectively increased intraoperative oozing was observed. En bloc resection of the right hepatic division, including the mass and CVC, was completed without intraoperative complications, and macroscopically complete resection was achieved (Figure 7). The cranial caval and femoral venous pressures were 0 and 11 mmHg, respectively, and the femoral arterial pressure was 131/104/93 mmHg (systolic/mean/diastolic pressure) after en bloc resection. The operative time was 120 min.

**FIGURE 6:**
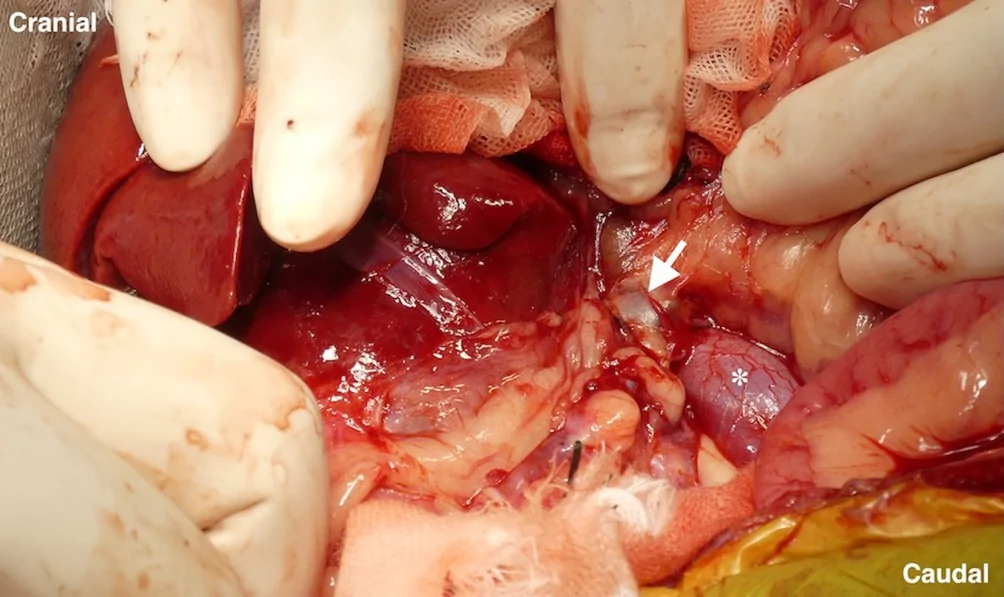
Intraoperative findings of the second‐stage surgery in dog 2. White arrow indicates the hydraulic occluder (HO). Asterisk indicates the caudal vena cava (CVC). Severe adhesions were present around the HO system.

**FIGURE 7:**
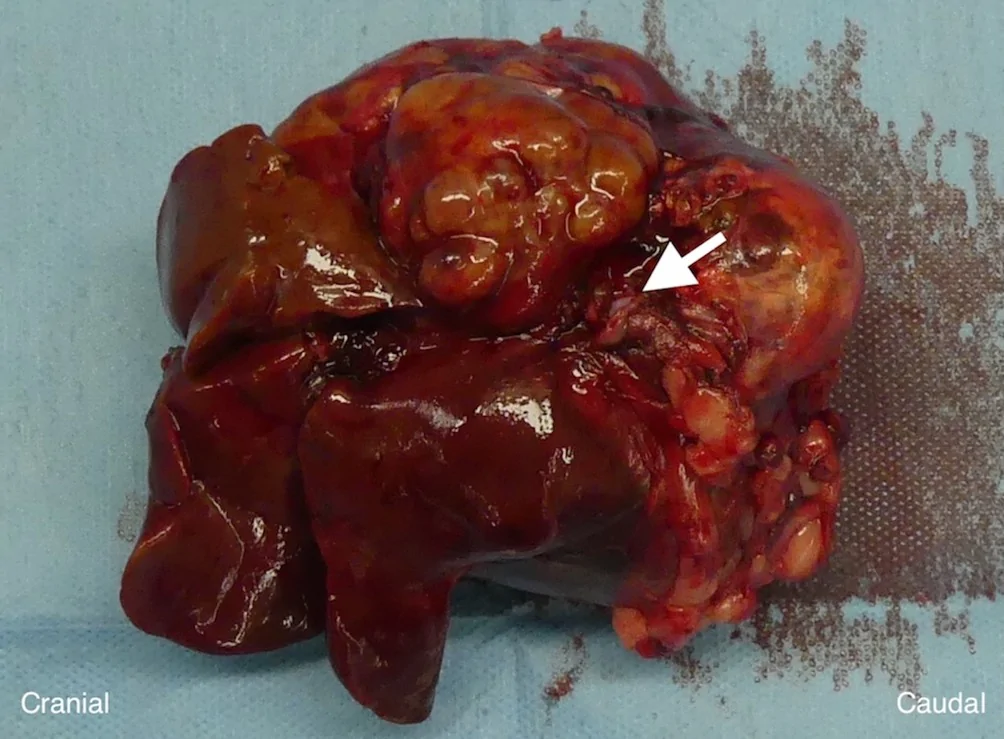
The resected mass of dog 2. White arrow indicates the caudal resection stump of the caudal vena cava (CVC).

Recovery from anesthesia was uneventful. A 100‐mL perioperative blood transfusion was administered because of the preoperative PCV trend and anticipated hemorrhage risk associated with right divisional hepatectomy, segmental CVC resection, and severe adhesions. The mass weighed 350 g (3.7% bodyweight). HCC with complete resection was confirmed histopathologically. Peritoneal effusion was observed from the next day after surgery, and then gradually decreased and disappeared by postoperative day 4. No hindlimb edema was observed during hospitalization. Additionally, postoperative BUN (20 mg/dL) and Cr (0.8 mg/dL) were maintained within the reference intervals.

Bodyweight decreased to 7.1 kg compared with the preoperative value; however, the owner reported normal appetite and activity, and the dog was discharged on postoperative day 7 after the second‐stage surgery. Skin sutures were removed on postoperative day 13. At that time, AST (14 U/L) and ALT (49 U/L) were within the reference intervals, whereas ALP (1121 U/L) and GGT (43 U/L) remained increased. On postoperative day 23, AST (15 U/L) and ALT (57 U/L) remained within the reference intervals, and ALP (259 U/L) and GGT (8 U/L) had decreased. On postoperative day 61 (last hospital follow‐up day), bodyweight had increased to 7.9 kg; AST (19 U/L), ALT (91 U/L) and GGT (4 U/L) remained within the reference intervals, whereas ALP (355 U/L) remained above the reference intervals.

After follow‐up at our hospital was concluded, care was returned to the referring veterinarian. Subsequent follow‐up information obtained from the owner and referring veterinarian indicated that thoracic and abdominal radiography and abdominal ultrasonography were performed at the referring hospital. Based on these examinations, the referring veterinarian reported no evidence of local recurrence or thoracic or abdominal metastasis. The dog died of unknown cause 2467 days after the second‐stage surgery (2517 days after the first evaluation).

## 4 DISCUSSION

Right divisional hepatectomy in dogs is technically challenging. Right‐sided tumors can be difficult to expose, and the main and accessory right hepatic veins course through the parenchyma before draining directly into the intrahepatic CVC.^3^, ^4^ These anatomic features can increase the risk of vascular trauma and inadequate hemostasis during dissection, particularly when a large right divisional HCC compresses or involves the CVC.^3^, ^4^ Although inflow occlusion may reduce hepatic arterial and portal venous inflow, hemorrhage from injured right hepatic veins or the intrahepatic CVC can remain substantial.^4^ Historically, right‐sided hepatic masses were associated with substantial intraoperative risk, including a reported 40% intraoperative mortality rate in one early series.^2^ However, in a more contemporary multi‐institutional study of 70 dogs undergoing right divisional hepatic lobectomy or partial lobectomy, no intraoperative deaths were reported. The perioperative mortality risk was 2.9%, suggesting lower perioperative mortality than previously reported.^11^ Nevertheless, intraoperative complications remained common in that study, and 21.4% of dogs required blood transfusion.^11^ Therefore, hemorrhage and vascular control remain clinically relevant, especially when the CVC is compressed or involved.^3^, ^11^ In the planned two‐stage strategy described here, complete pre‐renal CVC occlusion was induced to create alternative venous return. This strategy avoided individual dissection and ligation of the right hepatic veins. It also enabled en bloc resection including the involved caval segment. This approach may reduce the risk of hemorrhage related to vascular trauma and difficult hemostasis during right divisional hepatectomy.

Proceeding to second‐stage surgery requires both complete CVC occlusion and adequate collateralization. In an experimental model, complete occlusion was achieved over 2 weeks with small alternate‐day infusions.^10^ In the present cases, robust adhesions around the occluder suggested progressive local stenosis, and complete occlusion required two and three outpatient injections. A practical trigger for CT angiography is a sustained plateau of saphenous venous pressure despite additional inflation, with CT used to confirm occlusion and collateral formation.

Thrombosis is a key risk of planned caval occlusion. In this procedure, thrombus formation may reflect not only local venous stasis caused by progressive CVC occlusion, but also tumor‐associated hypercoagulability. Thromboelastography identified higher *G*‐values in dogs with surgically treated hepatocellular adenoma or carcinoma than in healthy control dogs.^12^ These findings suggest a hypercoagulable state associated with primary hepatocellular tumors. A large pre‐renal thrombus developed in dog 1, whereas no CVC thrombus was detected on preoperative CT in dog 2, which received clopidogrel during the staged occlusion period. The optimal perioperative antiplatelet strategy for this procedure remains unknown. In healthy dogs, clopidogrel inhibited adenosine diphosphate (ADP)‐induced platelet aggregation, and the duration of measurable antiplatelet effect ranged from >3 to >7 days after administration.^13^ Thus, discontinuing clopidogrel 2 days before surgery, as in dog 2, may not have eliminated its antiplatelet effect completely. However, the thrombotic risk associated with progressive CVC occlusion and possible tumor‐associated hypercoagulability must be balanced against the hemorrhagic risk of right divisional hepatectomy with segmental CVC resection. According to veterinary antithrombotic consensus recommendations,^14^ thrombotic risk should be stratified when antithrombotic therapy is discontinued. Continuation through invasive procedures is recommended for patients at high thrombotic risk receiving anticoagulant therapy. Discontinuation may be considered in patients with low‐to‐moderate thrombotic risk. In dog 2, clopidogrel was discontinued 2 days before the second‐stage surgery because substantial hemorrhage was anticipated, but no subjectively increased intraoperative oozing was observed. Because this finding is limited to a single dog and platelet function testing was not performed, no recommendation can be made regarding the optimal discontinuation interval. Future cases should individualize antithrombotic management according to thrombotic and hemorrhagic risks and consider coagulation assessment or platelet function testing when available.

The requirement for two operations imposes time and physiologic burden and introduces interval risks (progression, metastasis, rupture). Nevertheless, by limiting hemorrhage and facilitating complete resection, the strategy may improve the overall risk–benefit profile. Experimental data suggest the occlusion period could be shortened, potentially reducing interval events.^10^ Although the survival impact of margin status in canine HCC is debated, longer survival with complete resection has been reported.^7^, ^8^ In this series, survival was nearly 1 year and more than 6 years after complete resection. Larger clinical studies are needed.

In the two dogs reported here, staged pre‐renal CVC occlusion was feasible and permitted planned en bloc right divisional hepatectomy with segmental CVC resection after CT confirmation of complete caval occlusion and collateral venous return. However, these two cases do not establish repeatability or define optimal candidate selection, the ideal occlusion schedule, or tolerable saphenous venous pressure thresholds. This approach should therefore be considered only in carefully selected dogs. Potential candidates may include clinically stable dogs with a large right divisional hepatic mass, CT evidence of CVC compression or involvement, and no evidence of distant metastasis. Additional selection considerations include absent or inadequate pre‐existing collateral venous return, a disease‐free pre‐renal CVC segment suitable for occluder placement, and the ability to return for serial reassessment. Poor candidates may include dogs with rapid tumor progression, metastatic disease, poor anesthetic candidacy, or ongoing uncontrolled hemorrhage. Additional concerns include uncontrolled caudal venous hypertension during staged inflation, progressive renal dysfunction, failure to develop adequate collateral venous return, and inability to return for serial monitoring. Before proceeding to en bloc resection, complete pre‐renal CVC occlusion and adequate collateral venous return should be confirmed by CT.

Alternative local treatment modalities should also be considered for dogs with anatomically challenging hepatic tumors, particularly when complete surgical resection is not feasible or when operative risk is considered unacceptable. Nonsurgical or minimally invasive options for canine hepatocellular tumors have been reported.^15^, ^16^, ^17^, ^18^ These include percutaneous ultrasound‐guided microwave ablation,^15^ transarterial chemoembolization,^16^, ^17^ and three‐dimensional conformal radiation therapy.^18^ These modalities may provide local tumor control or palliation depending on tumor size, location, vascular involvement, treatment intent, and institutional availability. However, each requires specialized equipment and expertise, and currently available veterinary reports involve relatively small numbers of dogs. Therefore, their feasibility and expected benefit must be considered on a case‐by‐case basis. In the two dogs reported here, preoperative staging did not identify distant metastasis, and macroscopically complete en bloc resection, including removal of the involved caval segment, was considered achievable if alternative caudal venous return could be established. Because both tumors compressed or involved the CVC and no pre‐existing collateral venous return was present, staged pre‐renal CVC occlusion was selected to permit subsequent en bloc right divisional hepatectomy with segmental CVC resection.

In conclusion, planned gradual pre‐renal CVC occlusion before en bloc right divisional hepatectomy with segmental CVC resection has not been previously described in dogs. The two dogs in this report had right divisional HCC involving the CVC and lacked pre‐existing collateral venous return. In these two dogs, this approach was feasible and achieved macroscopically complete resection with transient morbidity. Staged pre‐renal CVC occlusion may establish alternative venous outflow and may reduce hemorrhagic risk in carefully selected dogs; however, additional cases are required to determine repeatability, optimal case selection, and safety.

## AUTHOR CONTRIBUTIONS

Asano K, DVM, PhD, Charter DJCVS: Conceived and designed the study. Ishigaki K, DVM, PhD and Asano K, DVM, PhD, Charter DJCVS: Performed the surgeries, managed perioperative care and follow‐up, drafted and revised the manuscript, and approved the final version.

## FUNDING INFORMATION

None.

## CONFLICT OF INTEREST STATEMENT

The authors declare no conflicts of interest related to this report.

## Data Availability

The data that support the findings of this study are available from the corresponding author upon reasonable request.
